# Clinical findings and prognosis of patients hospitalized for acute decompensated heart failure: Analysis of the influence of Chagas etiology and ventricular function

**DOI:** 10.1371/journal.pntd.0006207

**Published:** 2018-02-12

**Authors:** Caíque Bueno Terhoch, Henry Fukuda Moreira, Silvia Moreira Ayub-Ferreira, Germano Emilio Conceição-Souza, Vera Maria Cury Salemi, Paulo Roberto Chizzola, Mucio Tavares Oliveira, Silvia Helena Gelas Lage, Edimar Alcides Bocchi, Victor Sarli Issa

**Affiliations:** Heart Failure Department, Heart Institute (InCor) do Hospital das Clínicas da Faculdade de Medicina da Universidade de São Paulo, São Paulo, Brasil; Albert Einstein College of Medicine, UNITED STATES

## Abstract

**Aims:**

Explore the association between clinical findings and prognosis in patients with acute decompensated heart failure (ADHF) and analyze the influence of etiology on clinical presentation and prognosis.

**Methods and results:**

Prospective cohort of 500 patients admitted with ADHF from Aug/2013-Feb/2016; patients were predominantly male (61.8%), median age was 58 (IQ_25-75%_ 47–66 years); etiology was dilated cardiomyopathy in 141 (28.2%), ischemic heart disease in 137 (27.4%), and Chagas heart disease in 113 (22.6%). Patients who died (154 [30.8%]) or underwent heart transplantation (53[10.6%]) were younger (56 years [IQ_25-75%_ 45–64 vs 60 years, IQ_25-75%_ 49–67], *P* = 0.032), more frequently admitted for cardiogenic shock (20.3% vs 6.8%, *P*<0.001), had longer duration of symptoms (14 days [IQ_25-75%_ 4–32.8 vs 7.5 days, IQ_25-75%_ 2–31], *P* = 0.004), had signs of congestion (90.8% vs 76.5%, *P*<0.001) and inadequate perfusion more frequently (45.9% vs 28%, *P*<0.001), and had lower blood pressure (90 [IQ_25-75%_ 80–100 vs 100, IQ_25-75%_ 90–120], *P*<0.001). In a logistic regression model analysis, systolic blood pressure (*P*<0.001, OR 0.97 [95%CI 0.96–0.98] per mmHg) and jugular distention (*P* = 0.004, OR 1.923 [95%CI 1.232–3.001]) were significant. Chagas patients were more frequently admitted for cardiogenic shock (15%) and syncope/arrhythmia (20.4%). Pulmonary congestion was rare among Chagas patients and blood pressure was lower. The rate of in-hospital death or heart transplant was higher among patients with Chagas (50.5%).

**Conclusions:**

A physical exam may identify patients at higher risk in a contemporaneous population. Our findings support specific therapies targeted at Chagas patients in the setting of ADHF.

## Author summary

It is recognized that the clinical evaluation of patients remains the basis for the characterization of diseases, data interpretation, and patient care. However, incorporation of technological methods into clinical practice has challenged the way cardiologists’ value of history and clinical examination. The present study sought to analyze the importance of clinical findings in a contemporaneous cohort of patients admitted with decompensated heart failure. Our results indicate that the physical exam may identify patients at higher risk in a contemporaneous patient population, and that clinical presentation varies according to etiology—especially Chagas disease—and ventricular function. Our findings support the need of development of specific therapeutic approaches targeted at Chagas patients in the setting of acute decompensated heart failure, as they represent a more vulnerable population.

## Introduction

Advances in technologies applied to medical diagnosis have broadened medical understanding of patients and opened new possibilities for therapeutic interventions; however, it is recognized that the clinical evaluation of patients remains the basis for the characterization of diseases, data interpretation, and patient care.[1] Despite the value of clinical history to medical practice, incorporation of technological methods has challenged the way cardiologists’ value history and clinical examination.[2] Additionally, there has been concern regarding the possibility that clinical skills may be lost in face of the extensive technological evaluation currently available.[3]

This is particularly the case with heart failure,[4] a clinical syndrome that results from different processes affecting the cardiovascular system. As a heterogeneous entity,[5] various forms of categorization have been proposed to describe manifestations, predict prognosis, and identify patient groups that may benefit from specific interventions.[6] Most of the data used to elaborate such categorizations is obtained through history and clinical examination. In acute decompensated heart failure patients, assessment of prognosis in individual patients has been especially challenging due to the variability in the clinical course and presentation of the disease, along with the presence of different etiologies. Even though some findings, such as the presence of a third heart sound[7] and persistent congestion,[8] have been associated with a worse prognosis, detailed clinical features, and their associations with prognosis and therapy have not been fully evaluated during episodes of acute decompensation in recent series that included a broad spectrum of etiologies.[9,10,11,12]

This is particularly the case of Chagas cardiomyopathy, an etiology consistently associated with worse prognosis in the setting of chronic heart failure.[13] Interest in Chagas disease has grown not only due to its epidemiologic importance in both endemic and non-endemic areas,[14] but also due to the possibility of a specific pattern of treatment response.[15] Despite the availability of a large amount of data regarding the clinical presentation and prognosis of ambulatory patients with Chagas cardiomyopathy, information regarding its importance during episodes of acute decompensated heart failure is scarce.

In the present study, we hypothesized that clinical findings of patients with acute decompensated heart failure may vary according to the heart failure etiology and left ventricular function, and may contribute to the prognostic evaluation in a contemporaneous cohort of patients.

## Methods

### Ethics statements

The study was approved by the Institutional Ethics Committee for Research Project Analysis, which considered unfeasible and unnecessary to obtain formal consent from the patients studied.

### Objective

The aim of our study was to analyze the association between findings from history and physical exam with in-hospital prognosis in patients with acute decompensated heart failure, and analyze the influence of Chagas etiology and left ventricular function on patient presentation and prognosis.

### Study design

This was a prospective cohort study of patients admitted to the Heart Institute (InCor) of the Hospital das Clínicas da Faculdade de Medicina da Universidade de São Paulo (HC-FMUSP) with a diagnosis of acute decompensated heart failure. The first inclusion occurred in August 2013 and the last inclusion in February 2016. Patients were followed until hospital discharge and all patient data analyzed were anonymized.

### Patients

We included patients over 18 years of age admitted with a diagnosis of acute decompensated heart failure, irrespective of the ejection fraction. We excluded patients hospitalized for less than 24 hours and patients with cardiogenic shock or decompensated heart failure during the postoperative period after heart surgery.

### Variables

The data were obtained from medical records, including demographic information, epidemiological data, pathological history, reason for hospitalization, presence and duration of heart failure-related symptoms, etiologic diagnosis of heart failure or cardiomyopathy, physical examination data, electrocardiographic data, echocardiographic data, and major events during hospitalization, ie, death and heart transplantation.

### Definitions

The diagnosis of heart failure was made according to the Framingham criteria.[16] Acute heart failure was defined as new-onset decompensated heart failure or exacerbation of chronic heart failure that met the above criteria and required unplanned hospitalization.

The diagnosis of heart failure etiology was based on (1) typical clinical presentation for a given etiology; (2) presence of confirmatory tests, as indicated; (3) exclusion of other possible etiologies. The diagnosis of ischemic etiology was based on the presence of a history of myocardial infarction, percutaneous transluminal coronary angioplasty, previous coronary artery bypass surgery, stable angina pectoris with electrocardiographic changes, myocardial ischemia demonstrated by modified ergometric testing, and myocardial perfusion scintigraphy or coronary angiography showing ≥ 75% obstructions. The diagnosis of a Chagas etiology was based on positive serology for Chagas disease. The diagnosis of valvar etiology was based on a history of rheumatic heart disease or previous valve replacement surgery. The diagnosis of a hypertensive etiology was based on a history of severe hypertension and previous treatment with antihypertensive drugs, or evidence of left ventricular hypertrophy on electrocardiogram or echocardiogram in association with dilatation of the ventricular chambers.[17] The diagnosis of dilated cardiomyopathy was based on evidence of ventricular dilatation and systolic dysfunction by the different causes mentioned above. For the purpose of the present analysis, heart failure etiology was categorized in three groups: patients with Chagas cardiomyopathy, ischemic cardiomyopathy and those with dilated cardiomyopathy related to other conditions.

Patients were categorized according to the presence of symptoms and signs associated with congestion and/or inadequate perfusion. We considered as signs of congestion a history of orthopnea, jugular venous distention, rales, hepatojugular reflux, ascites, peripheral edema, and hepatomegaly; we considered symptomatic hypotension, cool extremities, impaired mentation, and oliguria to be signs of inadequate perfusion.

Patients were categorized according to hemodynamic profile at hospital admission. [18] We included in profile A, patients with no evidence of congestion or hypoperfusion; in profile B, patients with signs of congestion but adequate perfusion; in profile C, patients with signs of congestion and hypoperfusion; and profile L, patients with hypoperfusion but no signs of congestion. Patients were further categorized according to left ventricular ejection fraction measured by transthoracic echocardiography.

### Statistical analysis

Categorical variables are described as absolute value and percentage; continuous variables are described as median ± interquartile range 25–75%. For non-normal distribution of variables, the nonparametric Wilcoxon test was used, and for the normal distribution, the Student paired *t* test was used. Comparison of proportions between groups was performed with the X^2^ test. Multivariate analysis was performed with stepwise logistic regression. We included in the model variables with a *P* value in univariate analysis less than 0.1. *P* values less than 0.05 were considered significant. Statistical analysis was performed using SPSS for Windows version 11.0.

## Results

The study population consisted of 500 patients admitted with heart failure between August 2013 and February 2016 (Table 1); patients were predominantly male (61.8%), with a median age of 58 years (interquartile range [IQ] 47–66 years); main etiologies were dilated cardiomyopathy in 141(28.2%) patients, ischemic heart disease in 137 (27.4%), and Chagas heart disease in 113 (22.6%). The leading admission diagnoses were progressive heart failure (60.6%) and cardiogenic shock (12.4%); median left ventricular ejection fraction was 26% (IQ_25-75%_ 22–35); median creatinine at admission was 1.65 mg/dL (IQ_25-75%_ 1.23–2.34); and median brain natriuretic peptide was 1,086 pg/dL (IQ_25-75%_ 463–2,028). During hospital admission, 154 (30.8%) patients died and 53 (10.6%) underwent heart transplantation.

**Table 1:** Clinical characteristics of patients.

Clinical characteristics	N(%)/median(IQR_25-75_)
**Number of patients**	500
**Age (years)**	58 (47–66)
**Sex**	
Male	309 (61.8)
Female	191 (38.2)
**Heart failure etiology**	
Dilated cardiomyopathy	141 (28.2)
Ischemic heart disease	137 (27.4)
Chagas heart disease	113 (22.6)
Hypertension	56 (11.2)
Valvular heart disease	28 (5.6)
Others	25 (5.0)
**Admission diagnosis**	
Progressive heart failure	303 (60.6)
Cardiogenic shock	62 (12.4)
Arrhythmia/Syncope	53 (10.6)
Acute coronary syndrome	22 (4.4)
Infections	15 (3.0)
Others	45 (9.0)
**Duration of symptoms (days)**	10 (3–31)
**Previous history**	
Arterial hypertension	262 (52.4)
Atrial fibrillation	179 (35.8)
Diabetes Mellitus	156 (31.2)
Previous VT/VF	68 (13.6)
**LV ejection fraction (%)**	26 (22–35)
**Medications**	
Beta-blocker	397 (79.4)
ACE inhibitor/AT blocker	312 (62.4)
Spironolactone	269 (53.8)
Diuretics	377 (75.4)
Digoxin	115 (23)
Ivabradine	8 (1.6)
Warfarin	135 (27)
Inotropes	148 (29.6)
Creatinine (mg/dL)	1.65 (1.23–2.34)
Brain natriuretic peptide (pg/dL)	1086 (463–2028)
Implantable defibrillator	59 (11.8)

LV: left ventricle; VT: ventricular tachycardia; VF: ventricular fibrillation; ACE: angiotensin-converting enzyme

When clinical characteristics of patients were analyzed according to outcomes (Table 2), we found that, compared with patients discharged, patients who died or had a heart transplant during hospital stay were younger (median age 56 years [IQ_25-75%_ 45–64 versus 60 years, IQ_25-75%_ 49–67], respectively, *P* = 0.032), were more frequently admitted for cardiogenic shock (20.3% versus 6.8%, respectively, *P*<0.001), were less frequently admitted for arrhythmia or syncope (6.3% versus 13.7%, respectively, *P*<0.001), less frequently had a history of hypertension (45.9% versus 57%, respectively, *P*<0.018), had a longer duration of symptoms (14 days [IQ_25-75%_ 4–32.8 versus 7.5 days, IQ_25-75%_ 2–31], respectively, *P* = 0.004), more frequently had signs of congestion (90.8% versus 76.5%, respectively, *P*<0.001) and inadequate perfusion (45.9% versus 28%, respectively, *P*<0.001), and had lower blood pressure (90 [IQ_25-75%_ 80–100 versus 100, IQ_25-75%_ 90–120], respectively, *P*<0.001). These findings indicate the relevance of the admission diagnosis for the prognosis. Interestingly, we found no significant influence of gender, co-morbidities such as diabetes mellitus and atrial fibrillation in prognosis.

**Table 2:** Clinical characteristics of patients according to outcomes.

Clinical characteristics	Discharge% N(%)/median(IQR_25-75_)	Death/Transplant N(%)/median(IQR_25-75_)	P
**Number of patients**	293	207	
**Age (years)**	60 (49–67)	56 (45–64)	0.032
**Sex**			0.64
Male	184 (62.8)	125 (60.4)	
Female	109 (37.2)	82 (39.6)	
**Admission diagnosis**			<0.001
Progressive heart failure	178 (60.8)	125 (60.4)	
Cardiogenic shock	20 (6.8)	42 (20.3)	
Arrhythmia/Syncope	40 (13.7)	13 (6.3)	
Acute coronary syndrome	14 (4.8)	8 (3.9)	
Infections	8 (2.7)	7 (3.4)	
Others	33 (11.3)	12 (5.8)	
**Heart failure etiology**			0.072
Chagas Heart Disease	56 (19.1)	57 (27.5)	
Ischemic heart disease	81 (27.6)	56 (27.1)	
Dilated cardiomyopathy	156 (53.2)	94 (45.4)	
**Previous history**			
Hypertension	167 (57)	95 (45.9)	0.018
Diabetes mellitus	88 (30)	68 (32.9)	0.557
Atrial fibrillation	102 (34.8)	77 (37.2)	0.636
**Symptoms at admission**			
Orthopnea	134 (45.7)	109 (52.7)	0.146
NPD	121 (41.3)	99 (47.8)	0.17
Chest pain	91 (31.1)	52 (25.1)	0.16
Syncope	38 (3)	36 (17.4)	0.201
**Duration of symptoms (days)**	7.5 (2–31)	14 (4–32.8)	0.004
**Physical exam**			
Any sign of congestion	224 (76.5)	188 (90.8)	<0.001
Lower limbs edema	146 (49.8)	127 (61.4)	0.014
Pulmonary rales	131 (44.7)	99 (47.8)	0.524
Jugular distension	127 (43.3)	136 (65.7)	<0.001
Hepatomegaly	99 (33.8)	98 (47.3)	0.003
Ascites	50 (17.1)	60 (29)	0.002
Mitral systolic murmur	74 (25.3)	78 (37.7)	0.003
Tricuspid systolic murmur	25 (8.5)	20 (9.7)	0.751
Third heart sound	21 (7.2)	9 (4.3)	0.251
Inadequate perfusion	88 (28)	95 (45.9)	<0.001
Heart rate (bpm)	80 (68–100.5)	80 (68–96)	0.419
Systolic BP (mm Hg)	100 (90–120)	90 (80–100)	<0.001
Diastolic BP (mm Hg)	70 (60–80)	60 (56–70)	<0.001

bpm: beats per minute; BP: blood pressure; mm Hg: millimeters of mercury; NPD: nocturnal paroxysmal dyspnea

When these variables were analyzed in a logistic regression model, only systolic blood pressure (*P*<0.001, OR 0.97 [95% CI 0.96–0.98] per mmHg) and jugular distention (*P* = 0.004, OR 1.923 [95% CI 1.232–3.001]) remained as statistically significant variables (Table 3).

**Table 3:** Multivariable analysis of clinical findings associated with the occurrence of death or heart transplantation during hospital admission.

Variable	P	OR	CI 95%
Age	0.479	0.995	0.982–1.008
Lower limb edema	0.086	1.461	0.948–2.251
Jugular distension	0.004	1.923	1.232–3.001
Ascites	0.582	1.156	0.691–1.934
Hepatomegaly	0.626	0.894	0.571–1.401
Inadequate perfusion	0.226	1.296	0.852–1.973
Systolic mitral murmur	0.186	1.336	0.870–2.051
Systolic blood pressure	<0.001	0.970	0.960–0.980

OR: odds ratio; CI: confidence interval

### Analysis according to etiology

**Fig 1:**
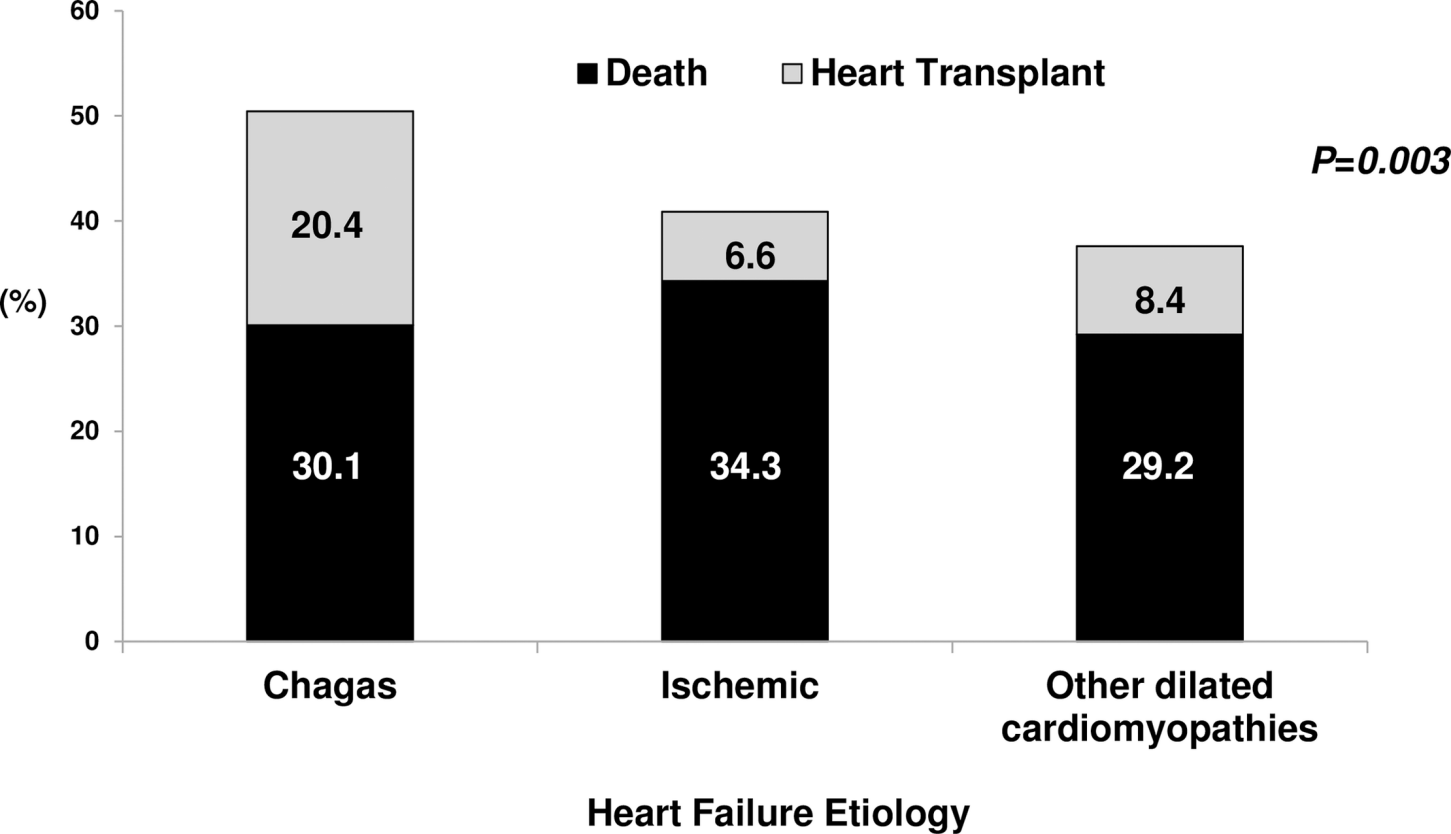
In-Hospital outcomes according to heart failure etiology.

When patients were analyzed according to etiology (Table 4), we found that their clinical characteristics differed regarding age, sex distribution, admission diagnosis, duration of symptoms, and findings on physical exam. Patients with Chagas cardiomyopathy and dilated cardiomyopathy were younger than patients with ischemic heart disease (median 56 years [IQ_25-75 45–63_]; 53.5 [41–64]; 63 [57–71], respectively; P<0.001) and had lower proportion of male patients (55.8%; 53.5% and 72.3% respectively, P = 0.01). Chagas patients were more frequently admitted for cardiogenic shock than patients with ischemic or dilated cardiomyopathy (15%; 11.7%; 11.6%, respectively; P<0.001). Furthermore, admission for syncope or arrhythmia was most frequent among Chagas patients (20.4%). Regarding findings on the physical exam, signs of pulmonary congestion were less frequently found among Chagas patients (pulmonary rales in 29.2%) compared with other etiologies. Admission blood pressure, however, was lowest among Chagas patients (systolic blood pressure 90 mm Hg [IQ_25-75_ 80–100]; diastolic blood pressure 61 mm Hg [IQ_25-75_ 55–72]). The distribution of in-hospital outcomes according to etiology showed that patients with Chagas cardiomyopathy had the highest rate of death or heart transplant (50.5%)(Fig 1); it is noteworthy the finding that patients with valvular heart disease had the highest in-hospital mortality (42.9%) and lowest frequency of heart transplantation (no patient received a transplant).

**Table 4:** Clinical characteristics of patients according to etiology.

Clinical characteristics	Chagas’ Disease N(%)/median (IQR_25-75_)	Ischemic Heart Disease N(%)/median (IQR_25-75_)	Other Dilated Cardiomyopathies N(%)/median (IQR_25-75_)	P
**Number of patients**	113	137	250	
**Age (years)**	56 (45–63)	63 (57–71)	53.5 (41–64)	<0.001
**Sex**				0.01
Male	63 (55.8)	99 (72.3)	147(58.8)	
Female	50 (44.2)	38 (27.7)	103 (41.2)	
**Admission diagnosis**				<0.001
Progressive heart failure	63 (55.8)	63 (46)	177 (70.8)	
Cardiogenic shock	17 (15)	16 (11.7)	29 (11.6	
Arrhythmia/Syncope	23 (20.4)	19 (13.9)	11 (4.4)	
Acute coronary syndrome	2 (1.8)	14 (10.2)	6 (2.4)	
Infections	2 (1.8)	8 (5.8)	5 (2.0)	
Others	6 (5.3)	17 (12.4)	22 (8.8)	
**Symptoms at admission**				
Orthopnea	54 (47.8)	54 (39.4)	135 (54)	0.023
NPD	47 (41.6)	50 (36.5)	123 (49.2)	0.046
Chest pain	30 (26.5)	41 (29.9)	72 (28.8)	0.837
Syncope	25 (22.1)	19 (13.9)	30 (12)	0.04
**Duration of symptoms (days)**	10 (3–31)	7 (1–30)	14(3–31)	0.035
**Physical exam**				
Lower limbs edema	59 (52.2)	68 (49.6)	146 (58.4)	0.215
Pulmonary rales	33 (29.2)	65 (47.4)	132 (52.8)	<0.001
Jugular distension	70 (61.9)	54 (39.4)	139 (55.6)	0.001
Hepatomegaly	55 (48.7)	35 (25.5)	107(42.8)	<0.001
Ascites	31 (27.4)	28 (20.4)	51 (20.4)	0.285
Mitral systolic murmur	43 (38.1)	29 (21.2)	80 (32)	0.011
Tricuspid systolic murmur	14 (12.4)	9 (6.6)	22 (8.8)	0.275
Third heart sound	7 (6.2)	5 (3.6)	18 (7.2)	0.37
Inadequate perfusion	51 (45.1)	37 (27)	89 (35.6)	0.012
Heart rate (bpm)	72 (60.5–88.5)	79 (65.5–98)	85 (70–103)	<0.001
Systolic BP (mmHg)	90 (80–100)	100 (88–120)	100 (83–110)	0.001
Diastolic BP (mmHg)	61 (55–72)	65 (60–80)	63 (60–77)	0.512

bpm: beats per minute; BP: blood pressure; mm Hg: millimeters of mercury; NPD: nocturnal paroxysmal dyspnea

**Fig 2:**
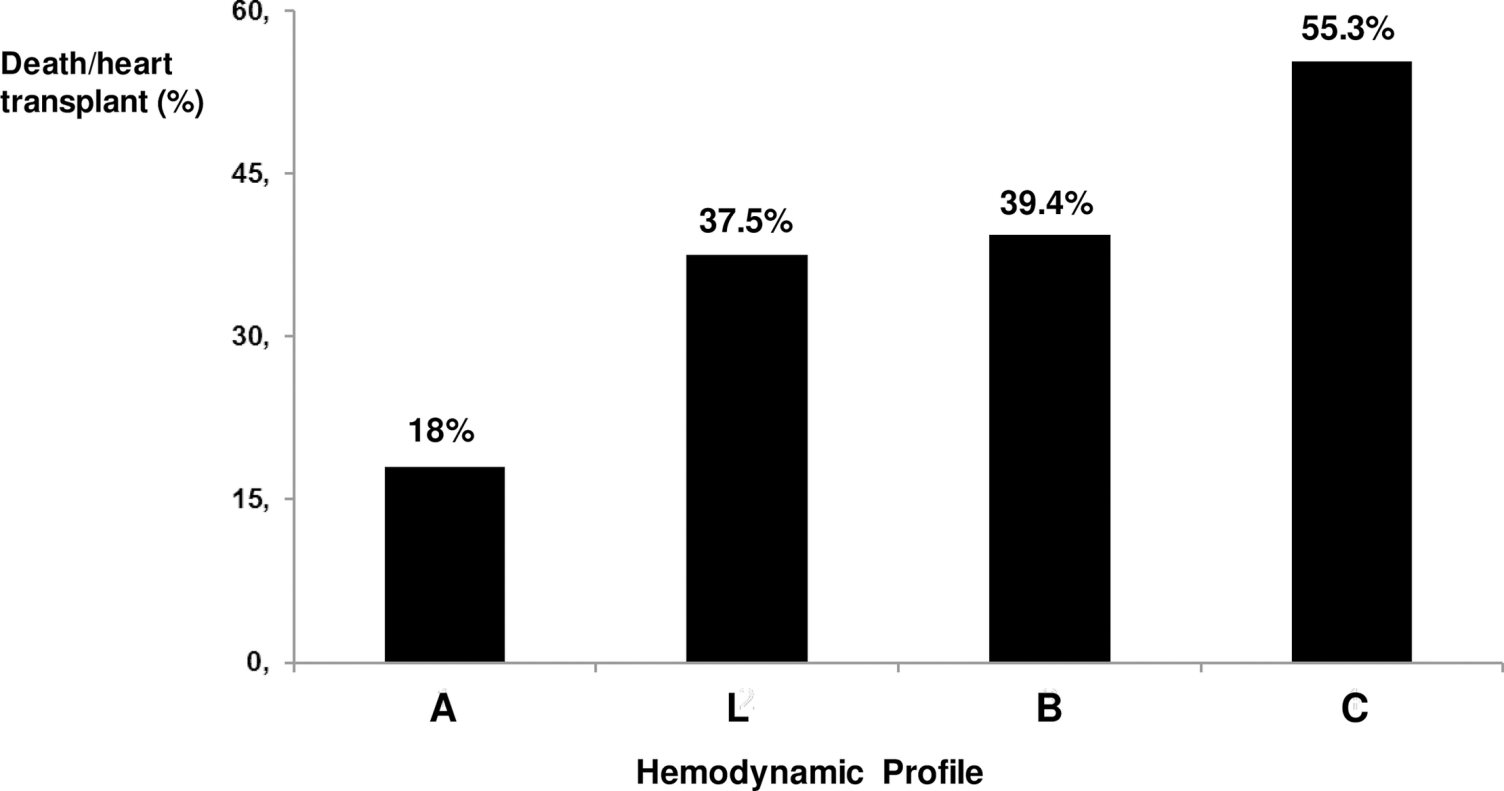
In-hospital prognosis according to hemodynamic profile. Profile A: patients with no evidence of congestion or hypoperfusion (dry-warm); profile L: patients with hypoperfusion without congestion (dry-cold); profile B: patients with congestion with adequate perfusion (wet- warm); profile C: patients with congestion and hypoperfusion (wet-cold).

**Fig 3:**
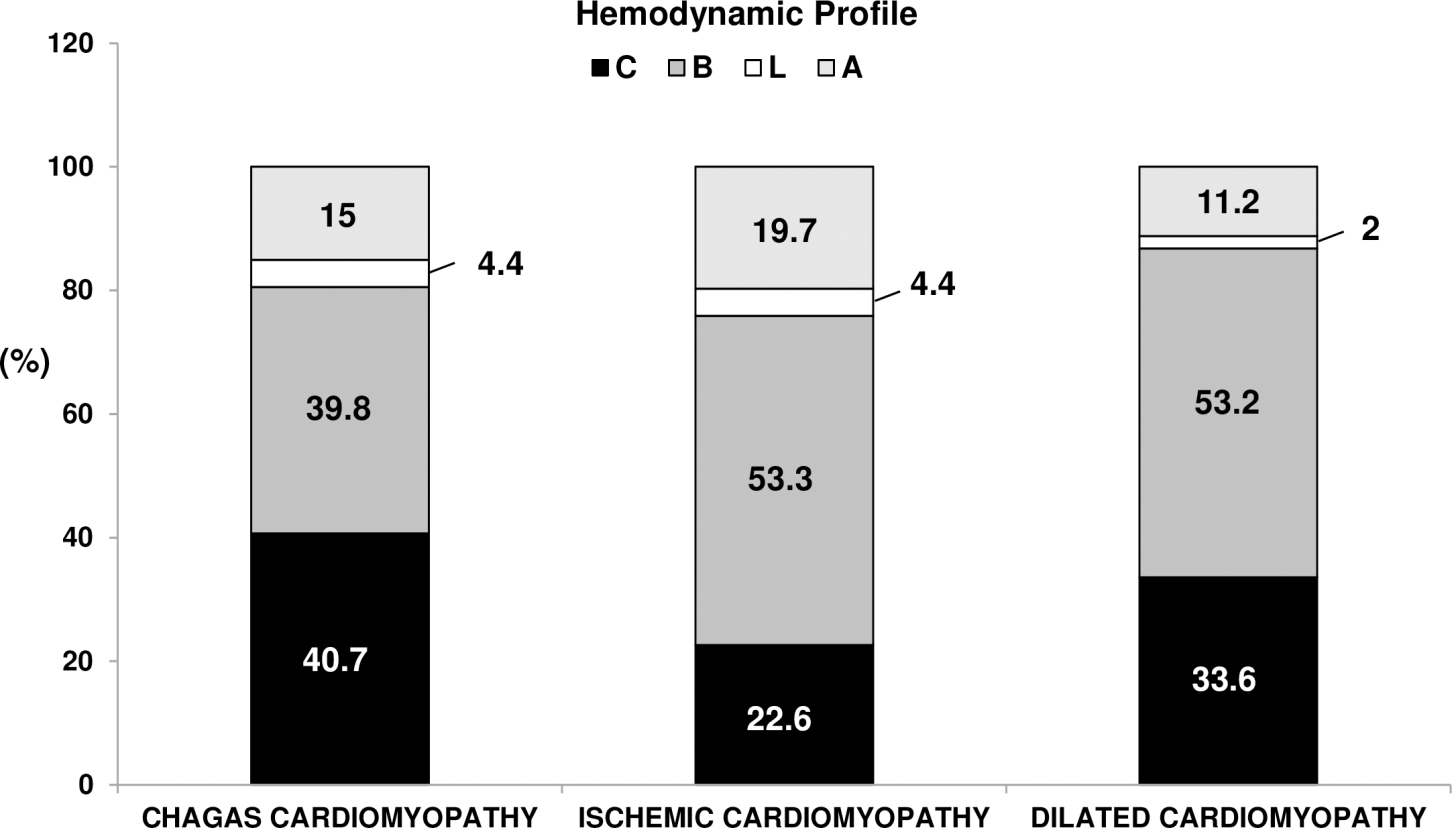
Distribution of the hemodynamic profile according to etiology.

We further analyzed patients according to the presence of specific hemodynamic profiles, based on clinical findings, as previously described: profile A (72 [14.4%] patients), profile B (251 [50.2%] patients), profile C (161 [32.2%] patients), and profile L (16 [3.2%] patients). Profiles B and C were associated with increased chance of heart transplant or death during hospital admission (39.4% and 55.3%, respectively) (Fig 2). The distribution of the hemodynamic findings varied according to heart failure etiology (Fig 3): profile C was more frequent in patients with Chagas as compared to other etiologies.

### Analysis according to ventricular function

When clinical characteristics of patients were analyzed according to left ventricular ejection fraction (LVEF) (Table 5), we found that patients with LVEF≥40%, compared with patients with LVEF<40%, were older (63 years [IQ_25-75%_ 55–76 versus 57 years, IQ_25-75%_ 45–64], respectively, *P*<0.001), were more frequently female (49.5% versus 45.5%, respectively, *P* = 0.014) and had a higher frequency of ischemic heart disease (34% versus 25.8%, respectively, *P*<0.001). Symptoms of orthopnea (34% versus 52.1%, respectively, *P* = 0.001) and nocturnal paroxysmal dyspnea (28.9% versus 47.6%, respectively, *P* = 0.001) were less frequent among patients with preserved LVEF compared with patients with reduced LVEF, as well as signs of right side congestion (73.2% versus 84.6%, respectively, *P* = 0.011); finally, blood pressure was higher among patients with preserved LVEF compared with patients with reduced LVEF (110 mm Hg, [IQ_25-75%_ 94–127 versus 92 mmHg, IQ_25-75%_ 80–110], respectively, *P*<0.001).

**Table 5:** Clinical characteristics of patients according to cardiac function.

Clinical characteristics	LV Ejection Fraction <40% N(%)/median(IQR_25-75_)	LV Ejection Fraction ≥40% N(%)/median(IQR_25-75_)	P
**Number of patients**	403	97	
**Age (years)**	57 (45–64)	63 (55–76)	<0.001
**Sex**			0.014
Male	260 (64.5)	49 (50.5)	
Female	143 (45.5)	48 (49.5)	
**Admission diagnosis**			0.174
Progressive heart failure	247 (61.3)	56 (57.5)	
Cardiogenic shock	56 (13.9)	6 (6.2)	
Arrhythmia/Syncope	39 (9.7)	14 (14.4)	
Acute coronary syndrome	16 (4)	6 (6.2)	
Infections	11 (2.7)	4 (4.1)	
Others	34 (8.4)	11 (11.3)	
**Heart failure etiology**			0.014
Chagas Heart Disease	101 (25.1)	12 (12.4)	
Ischemic heart disease	104 (25.8)	33 (34)	
Dilated cardiomyopathy	198 (49.1)	52 (53.6)	
**Previous history**			
Hypertension	202 (50.1)	60 (61.9)	0.042
Diabetes mellitus	123 (30.5)	33 (34)	0.542
Atrial fibrillation	140 (34.7)	39 (40.2)	0.346
**Symptoms at admission**			
Orthopnea	210 (52.1)	33 (34)	0.001
NPD	192 (47.6)	28 (28.9)	0.001
Chest pain	117 (29)	26 (26.8)	0.709
Syncope	63 (15.6)	11 (11.3)	0.341
**Duration of symptoms (days)**	12 (3–31)	7 (1–31)	0.149
**Physical exam**			
Any sign of congestion	341 (84.6)	71 (73.2)	0.011
Lower limbs edema	225 (55.8)	48 (49.5)	0.307
Pulmonary rales	184 (45.7)	46 (47.4)	0.821
Jugular distension	228 (56.5)	35 (36.1)	<0.001
Hepatomegaly	170 (42.2)	27 (27.8)	0.011
Ascites	95 (23.6)	15 (15.5)	0.101
Mitral systolic murmur	132 (32.8)	20 (20.6)	0.020
Tricuspid systolic murmur	34 (8.4)	11 (11.3)	0.428
Third heart sound	30 (7.4)	0 (0)	0.002
Inadequate perfusion	153 (38)	24 (24.7)	0.018
Heart rate (bpm)	81 (68–100)	78 (68–95)	0.198
Systolic BP (mm Hg)	92 (80–110)	110 (94–127)	<0.001
Diastolic BP (mm Hg)	60 (57–72)	70 (60–80)	0.004

bpm: beats per minute; BP: blood pressure; mm Hg: millimeters of mercury; NPD: nocturnal paroxysmal dyspnea

## Discussion

The present study sought to analyze the importance of clinical findings in a contemporaneous cohort of patients admitted with decompensated heart failure. The strengths of our study are based, first, on the finding that clinical characteristics of patients remain important for prognostic evaluation in a contemporaneous cohort of high-risk patients with advanced heart failure; second, clinical characteristics at presentation vary according to heart failure etiology, particularly among patients with Chagas disease, and according to left ventricular function, findings with significant clinical and therapeutic implications.

In the present cohort, median age was 58 years, patients were predominantly male (61.8%), and there was a high proportion of Chagas etiology (22.6%). It is important to note that in-hospital mortality was high (30.8%). These findings markedly contrast with reports from other authors. Data from the ADHERE registry that included patients from the United States showed a mean age of 72 years, a higher prevalence of female patients, and ischemic heart disease as the main etiology. Significantly, the in-hospital mortality reported was 4%.[19] Similarly, data from a European registry reported mean age 70±13 years and a predominance of male patients. The total in-hospital mortality rate was 3.8%.[20] Data from the Brazilian Registry of patients with decompensated heart failure reported a mean age of 64 years, a predominance of male patients, and in-hospital mortality of 12.6%.[21] Therefore, it should be noted that our study included a relatively young population with advanced heart failure. Possible reasons for these discrepancies are the inclusion of a high proportion of patients with Chagas heart disease that tend to affect a younger population compared with other forms of heart disease, especially ischemic heart disease.[22] Even though few other studies reported on the comparative outcomes of patients with Chagas heart disease and other etiologies, data from studies with patients with chronic heart failure indicate that Chagas patients have a worse prognosis compared with patients with hypertensive and ischemic heart disease[13,23,24] which may have contributed to the excessive mortality we have found. In the setting of decompensated heart failure, a recent study compared the prognosis of patients with Chagas cardiomyopathy to that of patients with other etiologies; no difference was found regarding in-hospital mortality, but Chagas patients had a higher rate of hospital readmission.[25] Additionally, our center is a tertiary hospital dedicated to cardiology that treats patients with advanced heart failure, with a higher expected mortality compared with that in community hospitals. In this sense, it is remarkable that a third of our patients received inotropes during their hospital stay.

We found that clinical findings at admission could identify patients with a worse prognosis during hospital stay, in particular, presence of cardiogenic shock, low arterial blood pressure and the presence of jugular distension. These findings are in accordance with previous reports and point to the importance of clinical examination of patients with acute decompensated heart failure. In the ADHERE registry, the presence of systolic blood pressure under 125 mm Hg was associated with a worse prognosis in patients with reduced ejection fraction, as well as in patients with preserved ejection fraction. Other clinical variables associated with prognosis in other studies were heart rate, dyspnea at rest, age, diabetes, and ischemic etiology.[26,27]

We found that the clinical presentation of patients was markedly influenced by heart failure etiology. Specifically, Chagas patients had the highest proportion of hospital admissions for cardiogenic shock (15%) and arrhythmia (20.4%), were more hypotensive, and had a higher proportion of patients with signs of right ventricular heart failure such as ascites (27.4%), hepatomegaly (48.7%) and jugular distension (61.9%). These findings suggest that right ventricular dysfunction may be more frequent in Chagas patients. Another study reported the presence of lower limb edema in 94.6% and of jugular engorgement of 48.6% in 37 Chagas patients as compared to 85.9% and 37.4%, respectively, in 99 patients with other etiologies; the differences were not statistically significant, what might be partially explained by the relatively small number of patients.[25] The finding of right ventricular dysfunction has been consistently described among patients with Chagas cardiomyopathy with and without heart failure.[28,29,30] Previous authors have described that pulmonary congestion is a rare phenomenon among Chagas patients and with a mild presentation.[31,32] Mechanisms related to this finding are largely unknown, but may be related to lower arterial blood pressure, autonomic dysfunction or modifications in left ventricular compliance.

Interestingly, when patients were categorized according to hemodynamic profile, Chagas had a higher proportion of patients with the C profile. Few other studies explored the association between heart failure etiology and clinical findings. A previous study[25] that included patients with Chagas heart disease found a higher occurrence of cardiogenic shock or arrhythmia during hospitalization, lower blood pressure, and a higher proportion of signs of right ventricular heart failure compared with other etiologies. To the best of our knowledge, no other study has explored the hemodynamic profile of Chagas patients in episodes of acute decompensation, a finding with significant therapeutic implications.

We found that the outcomes of patients were also markedly influenced by heart failure etiology and hemodynamic profile. Specifically, Chagas patients had the lowest proportion of hospital discharge, and the highest proportion of cardiac transplant when compared to other etiologies; therefore the worse prognosis of these patients is more related to the necessity of heart transplantation due to the severity of decompensation, reflected on clinical findings, than death during hospitalization. Few other studies explored the association between heart failure etiology and outcomes, and previous comparisons between ischemic versus nonischemic heart failure found lower survival in patients with ischemic heart disease. [33,34,35] In this respect, other studies have reported a worse prognosis among Chagas patients admitted with decompensated heart failure, including excessive mortality and higher rates of readmission.[25,36] Our data confirm that Chagas patients do have a worse prognosis during episodes of acute decompensated heart failure. Different mechanisms have been proposed for the worse prognostic found in patients with Chagas, and most data is derived from patients with chronic heart failure.[37] These mechanisms may include increased rate of right ventricular dysfunction, ventricular arrhythmias and conduction abnormalities as well and thromboembolic events[38,39]

### Conclusions

Taken together, our findings indicate that the physical exam may identify patients at higher risk in a contemporaneous patient population. Our findings reinforce the need of specific therapeutic approaches targeted at Chagas patients in the setting of acute decompensated heart failure, as they represent a more vulnerable population.

### Limitations

Some limitations of the present study should be acknowledged; we included a limited number of patients compared with other cohorts reported, and we did not include consecutive patients; therefore, we cannot exclude the possibility of selection bias. Additionally, as clinical data was obtained from medical records, heterogeneity regarding information from anamnesis and physical examination cannot be excluded. Prognostic information of patients was limited to the admission period, and our data may not be applicable to longer-term follow-up. Finally, some particular characteristics of our population, specifically the younger age and high proportion of patients with Chagas disease, may hinder the applicability of our results to other populations.
